# THE ECONOMIC IMPACT OF MENOPAUSE: A MULTIMODE RESEARCH PROJECT

**DOI:** 10.1093/geroni/igad104.1921

**Published:** 2023-12-21

**Authors:** Jennifer Sauer, Laura Mehegan, Alicia Williams, Aisha Bonner-Cozad, Cheryl Lampkin, Monica Ekman

**Affiliations:** AARP, Washington, District of Columbia, United States; AARP, Washington, District of Columbia, United States; AARP, Washington, District of Columbia, United States; AARP, Washington, District of Columbia, United States; AARP, Washington, District of Columbia, United States; AARP, Washington, District of Columbia, United States

## Abstract

In late 2022, AARP embarked on a robust research program to explore the economic impact of the menopause transition (perimenopause, menopause, and post-menopause) on affected individuals. Other research found that some women incur substantial costs to ameliorate adverse menopausal symptoms such as hot flashes, night sweats, brain fog, and mood swings. Additionally, some women experience symptoms severe enough to interfere with their job causing them to reduce their hours or leave their jobs completely. The first phase of AARP’s research included six online focus groups with US women aged 40 and older who were segmented by their stage of menopause (perimenopause, post-menopause). Resulting themes from the focus groups support what previous researchers have concluded that some individuals: (1) experience symptoms that disrupt their lives, (2) have treatments that are not necessarily discussed by a healthcare provider or covered by insurance leading to direct consumer costs, (3) consider leaving their jobs due to the severe and disruptive symptoms, and (4) feel their employers are not equipped to accommodate their needs. These focus group findings informed survey research among a representative sample of women aged 40 and older as well as an employer survey to explore any existing workplace policies that may benefit women experiencing the menopausal transition. Finally, all research results combined inform an information campaign to assist individuals who are navigating this stage of life.

